# Efficient and accurate *KRAS* genotyping using digital PCR combined with melting curve analysis for ctDNA from pancreatic cancer patients

**DOI:** 10.1038/s41598-023-30131-y

**Published:** 2023-02-21

**Authors:** Junko Tanaka, Tatsuo Nakagawa, Kunio Harada, Chigusa Morizane, Hidenori Tanaka, Satoshi Shiba, Akihiro Ohba, Susumu Hijioka, Erina Takai, Shinichi Yachida, Yoshio Kamura, Takeshi Ishida, Takahide Yokoi, Chihiro Uematsu

**Affiliations:** 1 Center for Digital Services - Healthcare, Research & Development Group, Hitachi, Ltd., 1-280, Higashi-Koigakubo, Kokubunji, Tokyo 185-8601 Japan; 2 Department of Hepatobiliary and Pancreatic Oncology, National Cancer Center Hospital, 5-1-1 Tsukiji, Chuo-ku, Tokyo 104-0045 Japan; 3 Department of Cancer Genome Informatics, Osaka University Graduate School of Medicine, 2-2 Yamadaoka, Suita, Osaka 565-0871 Japan; 4 Department of Otorhinolaryngology-Head and Neck Surgery, Osaka University Graduate School of Medicine, 2-2 Yamadaoka, Suita, Osaka 565-0871 Japan; 5 Division of Genomic Medicine, National Cancer Center Research Institute, 5-1-1 Tsukiji, Chuo-ku, Tokyo 104-0045 Japan

**Keywords:** Biological techniques, Biotechnology, Molecular biology, Oncology

## Abstract

A highly sensitive and highly multiplexed quantification technique for nucleic acids is necessary to predict and evaluate cancer treatment by liquid biopsy. Digital PCR (dPCR) is a highly sensitive quantification technique, but conventional dPCR discriminates multiple targets by the color of the fluorescent dye of the probe, which limits multiplexing beyond the number of colors of fluorescent dyes. We previously developed a highly multiplexed dPCR technique combined with melting curve analysis. Herein, we improved the detection efficiency and accuracy of multiplexed dPCR with melting curve analysis to detect *KRAS* mutations in circulating tumor DNA (ctDNA) prepared from clinical samples. The mutation detection efficiency was increased from 25.9% of the input DNA to 45.2% by shortening the amplicon size. The limit of detection of mutation was improved from 0.41 to 0.06% by changing the mutation type determination algorithm for G12A, resulting in a limit of detection of less than 0.2% for all the target mutations. Then, ctDNA in plasma from pancreatic cancer patients was measured and genotyped. The measured mutation frequencies correlated well with those measured by conventional dPCR, which can measure only the total frequency of *KRAS* mutants. *KRAS* mutations were detected in 82.3% of patients with liver or lung metastasis, which was consistent with other reports. Accordingly, this study demonstrated the clinical utility of multiplex dPCR with melting curve analysis to detect and genotype ctDNA from plasma with sufficient sensitivity.

## Introduction

Cell-free DNA (cfDNA) refers to small DNA fragments released from apoptotic and necrotic cells into body fluids, such as blood, lymph, and urine. Specifically, cfDNA released from tumor cells is called circulating tumor DNA (ctDNA)^1–5^. Because ctDNA reflects the state of malignancy, it is a remarkable marker that provides a less invasive approach to predict and evaluate the response to chemotherapy and to monitor disease recurrence. To increase the clinical utility of ctDNA for cancer diagnosis, quantification techniques with both high sensitivity and high multiplexing are needed.

Although the amount of ctDNA released into the blood varies depending on the cancer type, tumor burden, and metastasis, ctDNA is generally only a very small fraction of cfDNA. In particular, the mutation frequency is less than 1% in early-stage cancers^3,5^. Thus, a highly sensitive quantification technique is required to detect tiny amounts of ctDNA in the blood. The treatment efficacy of molecular targeted drugs can be predicted and evaluated by measuring drug-sensitizing and drug-resistance mutations. Molecular targeted therapy has greatly advanced in the field of treatment for non-small cell lung cancer (NSCLC), and *EGFR* is one of the best-studied molecular targets driving NSCLC^6–9^. Treatment with *EGFR* tyrosine kinase inhibitors (TKIs) led to significant responses in patients with *EGFR* exon 19 deletion and L858R mutations, whereas T790M mutations caused resistance to EGFR TKI treatment. To guide therapy, many mutations relevant to the treatment should be tested in a multiplex assay.

Quantitative real-time PCR, next-generation sequencing (NGS), and digital PCR (dPCR) are used to measure ctDNA. Quantitative real-time PCR is a low-cost and simple method for quantification, and the concentration of the target is calculated using a standard curve^10^. NGS is able to quantify many mutations at the same time, but the cost of the test presents a challenge. Moreover, the quantification of low-frequency mutations is not reproducible between instruments or facilities^11,12^. dPCR is used in liquid biopsy to quantify nucleic acids with high sensitivity^13,14^. Direct quantification of nonmutated and mutated sequences using dPCR provides information on the percentage representation of mutated sequences. In dPCR, the samples are diluted and partitioned into many separate compartments, each containing either one copy or zero copies of the target gene. PCR is performed in each compartment, and the endpoint fluorescence is measured to determine if the compartment is positive or negative. Then, the target gene in the sample can be digitally counted. Conventional dPCR discriminates among multiple targets through the color of the fluorescent dyes of the probe. Thus, the overlap of the spectrum of fluorescent dyes places a limit on multiplexity. To increase the multiplexity beyond the number of different fluorescent dyes, we developed a dPCR technique combined with melting curve analysis^15,16^. We demonstrated the simultaneous discrimination of 10 different genotypes by combining the colors of the fluorescent dyes and the melting temperatures.

This report describes the first demonstration of multiplex dPCR with melting curve analysis applied to detect mutations in ctDNA prepared from clinical plasma samples. We chose the 7 most common mutations (G12D, G12R, G12V, G13D, G12A, G12C, and G12S) in codons 12 and 13 of the *KRAS* oncogene for this demonstration. *KRAS* mutations are one of the most common driver mutations in cancer. More than 90% of patients with pancreatic ductal adenocarcinoma (PDAC) have *KRAS* mutations^17^. Because *KRAS* mutations are known to make *EGFR* inhibitors less effective, *KRAS* mutations have been tested as a negative selection to predict response to EGFR TKIs in colorectal cancer patients^18^. *KRAS* was previously thought to be an undruggable target due to the high affinity of *KRAS* for GTP, which is abundant in human cellular tissues, and the lack of deep binding pockets for specific small molecule inhibitors^19^. However, in 2021, sotorasib was finally approved by the FDA as a molecular target for the G12C mutation of *KRAS*, which is present in 5–15% of NSCLCs^20^. In addition to sotorasib, molecular targeted drugs and vaccines targeting G12C and other *KRAS* mutations are being developed^19^. Since the sensitivity to drugs targeting these *KRAS* mutations varies depending on the mutation type, the need for *KRAS* genotyping to enable treatment selection will increase in the future. In this study, *KRAS* genotyping by dPCR combined with melting curve analysis was applied to ctDNA extracted from the plasma of pancreatic cancer patients. In our previous report on *KRAS* genotyping combining dPCR and melting curve analysis^16^, there were issues with the detection efficiency of short fragmented cfDNA and the discrimination accuracy of genotyping. To solve these problems, we increased the detection efficiency of cfDNA by reducing the amplicon size and improved the discrimination accuracy of genotyping by changing the mutation type determination algorithm. Our multiplex dPCR method determined the *KRAS* genotype of the ctDNA in the plasma samples. The mutation frequency correlated well with that measured by conventional dPCR, which can measure only the total frequency of *KRAS* mutants. Therefore, multiplex dPCR with melting curve analysis was shown to be capable of genotyping and quantification in clinical samples.

## Results

### Overall workflow of dPCR with melting curve analysis

**Figure 1:**
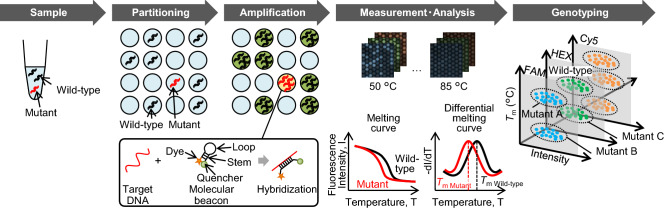
Procedure of dPCR combined with melting curve assay. Samples including target DNA were partitioned into microwells, and asymmetric PCR was performed with molecular beacons in the wells. Fluorescence images depending on temperature were captured with three-color filters, and the melting curves were analyzed for each well. Genotyping was performed according to fluorescence intensity, dye color, and *T*_m_.

We have previously reported our dPCR measurement system combined with melting curve analysis^16^. Figure 1 shows the overall workflow of our dPCR measurement system. dPCR measurement with melting curve analysis consisted of five steps: preparation of the reaction solution, partitioning of the reaction solution into microwells, PCR, fluorescence measurement and melting curve analysis, and genotyping. First, in the reaction solution preparation step, the specimen, enzymes, primers, and probes were mixed. For the probe, we used a molecular beacon^21^, which is not degraded by polymerase during PCR, instead of hydrolysis probes such as TaqMan probes. The molecular beacon had a stem‒loop structure, as shown in Fig. 1. The loop moiety was complementary to the target DNA, and a fluorescent dye and a quencher were bound to each end of the stem. In the partitioning step of the reaction solution, the PCR solution was partitioned into a silicon chip (QuantStudio 3D chips from Thermo Fisher Scientific) with up to 2 × 10^4^ wells. In the PCR step, the target DNA in the wells was amplified by asymmetric PCR to obtain single-stranded amplicons complementary to the molecular beacon probe^22,23^. If the wells contained target DNA, the molecular beacons hybridized to the amplified target, and the fluorescence intensity of the wells became higher than that of wells without the target DNA. In the fluorescence measurement and melting curve analysis step, the chip was placed on the temperature control stage of the custom-made instrument, and fluorescence images were captured while controlling the temperature of the chip. The melting curve of each well was obtained by plotting the fluorescence intensity against the temperature of the chip, and the *T*_m_ was calculated by differentiating the melting curve. Finally, in the genotyping step, the genotype of the DNA in the wells was determined from the fluorescence intensity, the color of the probe dye, and the *T*_m_.

### Optimization of dPCR for detecting *KRAS* mutants in plasma

We have previously demonstrated the genotyping of *KRAS* and *BRAF* genes in genomic DNA standards using a dPCR measurement system combined with melting curve analysis^16^. However, there were issues with the detection efficiency of short fragmented cfDNA and the discrimination accuracy of genotyping. In this study, we optimized dPCR to demonstrate *KRAS* genotyping in short fragmented cfDNA extracted from the plasma samples of cancer patients. Furthermore, we improved the discrimination accuracy of genotyping by changing the mutation type determination algorithm. We describe the optimization of dPCR for detecting short fragmented cfDNA in this section and show the improvement of discrimination accuracy of genotyping in the next section.

Since cfDNA is fragmented to an average of 165 bp in blood^24^, the detection efficiency of cfDNA is higher when the amplicon size is shorter. Many studies on the detection of cfDNA have used primer sets with amplicon sizes of less than 100 bp^25–27^. To avoid amplifying the two pseudogenes (*KRASP1* and processed pseudogene *KRASP1*) that have high homology with the *KRAS* gene, we used a forward primer (blue arrow in Supplemental Fig. S1) located at the intron region that has no homology between *KRAS* and the pseudogenes in our previous report^16^, referring to Mancini et al.^28^. Although the amplification of the pseudogene was suppressed, the amplicon size was 103 bp, which was too long for cfDNA detection. In this study, we redesigned the primers to suppress the amplification of the pseudogene by using the mismatched bases between the *KRAS* gene and the pseudogene located near the mutations in codons 12 and 13 of the *KRAS* gene (purple arrows in Supplemental Fig. S1). The amplicon size was 66 bp, enabling efficient cfDNA detection.

Melting curves were analyzed by real-time PCR using the designed primers to evaluate whether the amplification of pseudogenes was suppressed. When the annealing temperature was 55 °C, the new primer set showed a small peak near 62 °C that was different from the wild-type *T*_m_ peak at 70 °C (Supplemental Fig. S2A). When the annealing temperature was 60 °C, the peak at 62 °C decreased (Supplemental Fig. S2B). Furthermore, when a nonfluorescent blocker that binds to processed *KRASP1* was added, the peak at 62 °C decreased as the blocker concentration increased (Supplemental Fig. S2C), suggesting that the peak at 62 °C was due to the interaction between the pseudogene and the probe for wild-type *KRAS*. When the genomic DNA standard was amplified by dPCR with the new primer set and blocker, several copies of pseudogenes were amplified by annealing at 55 °C. Annealing at 60 °C allowed the efficient amplification of *KRAS* and completely suppressed the amplification of the pseudogenes (data not shown).

**Figure 2:**
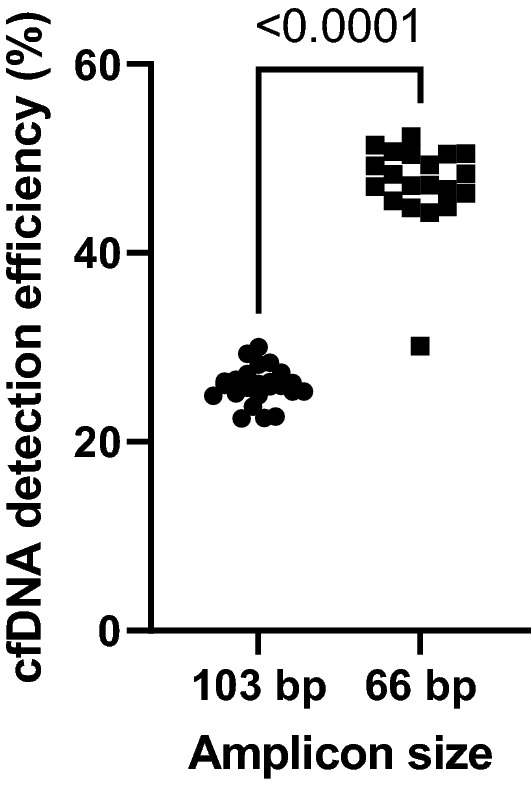
The relationship between amplicon size and cfDNA detection efficiency. The detection efficiency of cfDNA was improved significantly by shortening the amplicon size from 103 to 66 bp. N ≥ 20 for each primer set.

Then, we evaluated the cfDNA detection efficiency of dPCR combined with melting curve analysis using cfDNA standard samples of the *KRAS* gene. cfDNA standard samples were measured using the new primer set for the *KRAS* gene. After correction for Poisson distribution, 45.2 ± 4.7% of input cfDNA was detected (Fig. 2). Since the detection efficiency of the conventional primer was 25.9 ± 1.9%, the detection efficiency of cfDNA was improved by 1.7-fold by shortening the amplicon size from 103 to 66 bp. Note that the same detection efficiency is expected for the target *KRAS* mutations because the amplicon size of the mutations is the same as that of the wild-type.

### Genotyping of *KRAS* mutants

**Figure 3:**
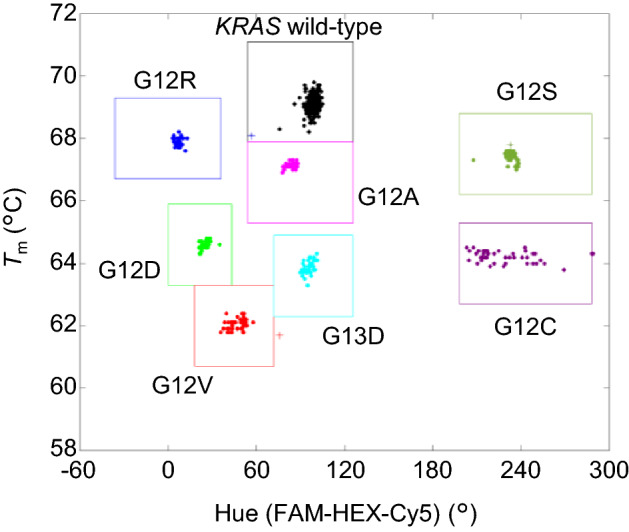
Genotyping results of the proposed eight-plex assay with the mixed sample of wild-type *KRAS* and G12A, G13D, G12R, G12D, G12V, G12S, and G12C mutants. Dots indicate a single positive sample of wild-type or mutants; plus signs indicate a double positive sample of wild-type and one mutant.

Figure 3 is a graph showing the genotyping results of the mixed genomic DNA standards. According to the analysis method in a previous report^16^, the horizontal axis represents the hue value calculated from the normalized intensities measured with the FAM, HEX, and Cy5 filters, and the vertical axis represents the *T*_m_ value. The genotyping window shown in Fig. 3 was manually chosen using the results of mixed samples, and the same window was used for all experiments. In addition, Supplemental Fig. S3 shows the genotyping results for various genomic DNA standards. The detected DNA groups were clearly clustered in the distribution of *T*_m_ and hue values. The genotyping results were consistent with the input samples.

Since the target DNA is partitioned into many microwells based on a Poisson distribution, most of the wells contain one or zero copies of the target DNA, while some wells contain two or more copies. Double-positive wells containing DNA of different genotypes can be distinguished from each other by their hue and *T*_m_ values. In Fig. 3 and Supplemental Fig. S3, wells containing both wild-type and mutant DNA are plotted with plus signs in the color of the mutant. For example, there is one plus sign on the right side of the G12V window in Fig. 3. This well was recognized to contain wild-type and G12V and is plotted outside the G12V window based on the intermediate hue value between the HEX-labeled probe for the wild-type and the FAM-labeled probe for G12V and on the *T*_m_ value of G12V calculated from the melting curve of FAM.

**Figure 4:**
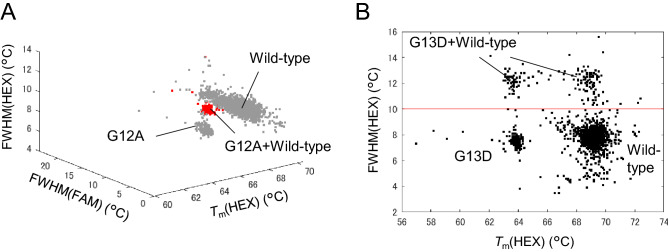
Discriminating between wells containing two different genotypes. (**A**) Double-positive wells containing wild-type and G12A and single-positive wells containing wild-type or G12A were discriminated by using the *T*_m_ values from the HEX filter and FWHM values of the differential melting curve measured from the FAM and HEX filters. (**B**) Double-positive wells containing wild-type and G13D and single-positive wells containing wild-type or G13D were discriminated by using the *T*_m_ values measured from the HEX filter and FWHM values of the differential melting curve measured from the HEX filter.

On the other hand, when two types of DNA detected by the same color probe were in the same well, it was difficult to identify the two types only by the *T*_m_ value calculated from the melting curve, and double positives were not determined in the previous report^16^. In this *KRAS* genotyping, wild-type, the G12A *KRAS* mutant and the G13D *KRAS* mutant were detected with HEX-labeled probes. Although the *T*_m_ peak values were not able to clearly distinguish the wells containing both the wild-type and the mutant from the wells containing either the wild-type or the mutant due to *T*_m_ variations, the peak width of the double-positive well was wider than that of the wells with a single target. Therefore, we used not only the *T*_m_ value but also the full width at half maximum (FWHM) of the differential melting curve to discriminate the double positives of G12A and G13D (Fig. 4). As a result, the deviations from the certified copy number of genome standards were reduced by the correction of double positive counts.

### Validation of the limit of detection

We evaluated the sensitivity of dPCR using melting curve analysis in *KRAS* detection. Cell-free DNA standard samples containing different frequencies of the G12D mutant were used for the sensitivity evaluation of G12D. We used fragmented wild-type genomic DNA standards spiked with the respective fragmented genomic DNA standards for the sensitivity evaluation of mutations other than G12D.

**Figure 5:**
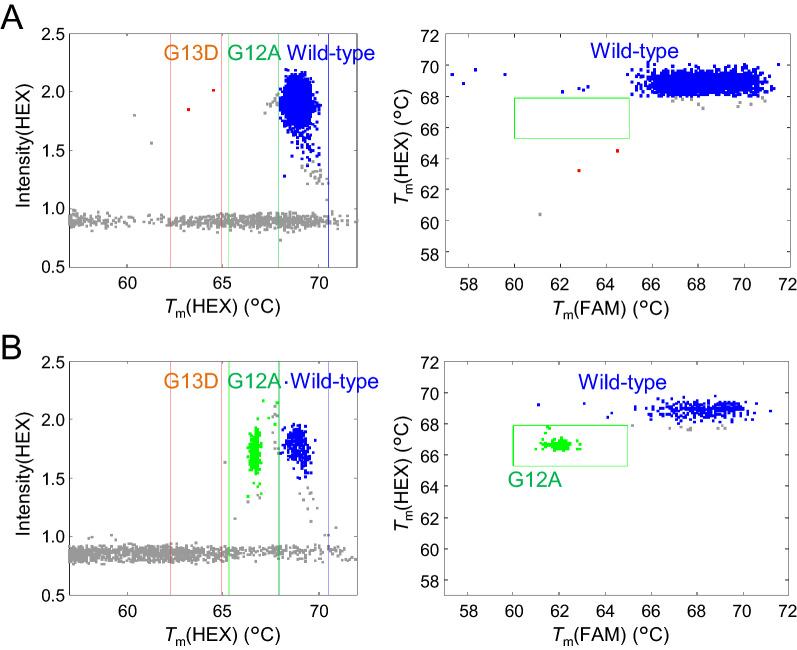
Genotyping of *KRAS* wild-type and G12A using the *T*_m_ values of FAM for each genome standard sample. (**A**) Wild-type, (**B**) G12A. Left, Scatter plot of normalized fluorescence intensity and *T*_m_ of each well with the fluorescent HEX filter. Right, scatter plot of the *T*_m_ of each well with the fluorescent FAM and HEX filters.

**Figure 6:**
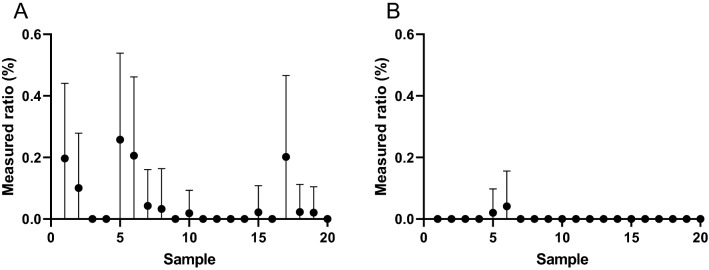
Percentage of wild-type samples misclassified as G12A before (**A**) and after (**B**) the addition of the *T*_m_ value calculated from the FAM filter as a parameter in the G12A determination. Error bars indicate Poisson error values. N = 20.

The sensitivity evaluation experiment revealed that the wild-type tends to form false positives for G12A. Both the wild-type and the G12A mutant were detected with HEX-labeled probes, and the *T*_m_ difference between the wild-type and G12A was small, approximately 2 °C. Thus, as shown in the left graph of Fig. 5A, the edge of the wild-type population can be counted as G12A even in the genomic standard sample containing only the wild-type when discrimination is performed by HEX fluorescence intensity and *T*_m_ value. We examined the relationship between the *T*_m_ values of wild-type and G12A calculated from fluorescence images of the FAM and HEX filters using the measured data of G12A genomic standard samples and found that G12A has a specific *T*_m_ value in the FAM filter, as shown in the right graph of Fig. 5B. This is because, as shown in Supplemental Table S1, G12A has a single-nucleotide mutation in the same location as G12D and G12V, which are detected by the FAM-labeled probe. Since the G12D probe had a high affinity for G12A, the G12D probe and the amplified G12A DNA hybridized in the wells, and their *T*_m_ values were observed with a FAM filter. According to this result, we added the *T*_m_ value calculated from FAM as a parameter to determine G12A. Figure 6 compares the percentage of wild-type standards (N = 20) incorrectly determined as G12A before and after the addition of the *T*_m_ value calculated from the FAM filter as a parameter to determine G12A. The percentage of wild-type samples misclassified as G12A was greatly reduced by the change in the G12A determination algorithm.

As shown in Supplemental Fig. S4, the input G12D ratio and the detected G12D ratio were proportional with a slope of 0.99, and the R^2^ correlation coefficient was 0.996. Supplemental Figure S5 also shows that the input and detected ratios were proportional in samples of other mutants, and the slope and R^2^ correlation coefficient of the fitted line were 0.9‒1.0 and > 0.99, respectively.

The limit of detection (LOD) of each mutant was calculated from the standard deviation and mean values measured with cfDNA standards consisting of 100% wild-type standards (N = 8), and the slope of the calibration curve for each mutation is shown in Supplemental Figs. S4 and S5. Table 1 shows the LOD of each *KRAS* mutant. For all mutations, the LODs were lower than 0.2%. The addition of the *T*_m_ value calculated from the FAM filter as a parameter in the determination of G12A decreased the LOD of G12A from 0.41 to 0.06%. The LODs for most mutants were less than 0.1%; however, the LODs of G13D and G12S were higher than 0.1%, possibly because G13D was detected by the same probe as the wild-type with a single mismatch, so when mutations other than those of the prepared probe occur in the wild-type DNA due to PCR errors, they are plotted in the window of G13D. Unfortunately, G13D has a two-nucleotide mismatch with the other probes, and it is difficult to introduce additional parameters for determination using different fluorescence information, as in G12A determination. In the detection of G12S, all wells in which G12S was detected were double positive for wild-type and G12S (Supplemental Table S2). Therefore, it is likely that the wild-type was mutated during amplification due to a PCR error, resulting in the amplification of the wild-type and G12S in the same well. The reason for this higher frequency of PCR errors in G12S compared to the other mutants may be related to the sequence characteristics of the polymerase^29^.

**Table 1:** LOD of each *KRAS* mutant measured by dPCR with melting curve analysis.

*KRAS* mutants	LOD (%)
G12R	0.03
G12D	0.07
G12V	0.04
G12A	0.06
G13D	0.13
G12S	0.17
G12C	0.03

### dPCR of ctDNA in plasma from PDAC patients

To demonstrate the possibility of detecting and quantifying tumor DNA directly from the plasma of PDAC patients, we performed multiplex analysis of *KRAS* in 46 plasma samples. After extracting and purifying cfDNA from the plasma of patients with advanced PDAC, we quantified the amount of cfDNA per ml of plasma using a fluorescent reagent for nucleic acid quantification. Additionally, the copy number of the *KRAS* gene was quantified using dPCR. As shown in Supplemental Fig. S6, the amount of cfDNA correlated with the copy number of the *KRAS* gene. By calculating the ratio of the detected copy number to the amount of cfDNA, the detection efficiency of cfDNA in plasma was determined. The cfDNA detection efficiency by dPCR was 66.2 ± 20.1%, which was higher and more varied than that of the cfDNA standard (45.2 ± 4.7%). The difference in detection efficiency is presumably because the fragment size of cfDNA in clinical samples varies from patient to patient and has a wide distribution, while the cfDNA standard has a relatively uniform fragment size because it is mechanically sheared genomic DNA extracted from engineered cell lines. We increased the detection efficiency for fragmented cfDNA by shortening the amplicon size.

**Figure 7:**
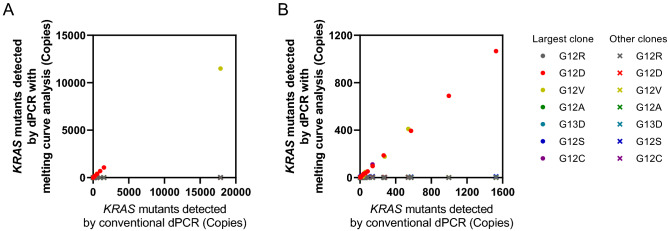
Comparison between the *KRAS* mutant copies detected by dPCR with melting curve analysis and conventional dPCR. (**B**) Shows enlarged low copy data of (**A**).

We compared dPCR combined with melting curve analysis and conventional dPCR by measuring cfDNA from PDAC patients. Figure 7 shows the comparison of the copy number of the detected *KRAS* mutations. Conventional dPCR is able to measure only the total frequency of *KRAS* mutants, and the genotype cannot be identified. In Fig. 7, the colors of the plots show the genotype observed by dPCR using melting curve analysis. As shown in Fig. 7, the copy number of one *KRAS* mutant correlated with the copy number of *KRAS* mutations detected by conventional dPCR (the R^2^ of the correlation was ≥ 0.99), while the other mutations were plotted near the x-axis due to the small number of copies observed. Multiple *KRAS* mutations were not observed at a high frequency in a single sample.

Supplemental Figure S7 and Supplemental Table S3 show the results of the comparison of variant allele frequencies (VAFs) detected by dPCR using melting curve analysis and conventional dPCR. In Supplemental Fig. S7, only the genotypes with the highest VAFs detected by dPCR using melting curve analysis are plotted for each clinical sample. The VAFs detected by dPCR using melting curve analysis and conventional dPCR showed a high correlation (the R^2^ of the correlation was ≥ 0.99). In samples #24 and #25, G12S mutants were detected by dPCR using melting curve analysis, but they were almost all double-positive wells for the wild-type and G12S (Supplemental Table S4).

## Discussion

Excluding the false positives of samples #24 and #25, 56.5% of all samples and 62.1% of stage IV samples were positive for *KRAS* mutations. Mutations in *KRAS* are among the first to occur during carcinogenesis and are observed in > 90% of pancreatic cancer patients^17^, but the positive rate in cfDNA has been reported to be lower. Takai et al. detected four *KRAS* mutations (G12R, G12D, G12V and G13D) by dPCR and found that 58.9% of stage IV samples were positive for *KRAS* mutations^26^. Sefrioui et al. detected seven *KRAS* mutations (G12R, G12D, G12V, G12A, G13D, G12S and G12C), the same as those detected in this study, by dPCR, and 65% of patients with advanced cancer were mutation positive^30^. These two reports of *KRAS* mutations detected by dPCR are nearly consistent with the positive rate for *KRAS* obtained in this study. Furthermore, Sugimoto et al. detected 16 different mutations in codons 12 and 13 of *KRAS* by dPCR and found that the positive rate was higher in liver or lung metastases than in peritoneal metastases^31^. As shown in Supplemental Fig. S8, *KRAS* mutations were detected in 8/17 samples (47.1%) of locally advanced cancer, 4/12 samples (33.3%) of peritoneal or lymph node metastasis, and 14/17 samples (82.3%) of liver or lung metastasis. Our data also indicated a higher positivity rate in liver or lung metastasis. Supplemental Fig. S8A–C shows the amount of cfDNA, the copy number of ctDNA, and the VAF for each metastatic state. The amounts of cfDNA and VAF were significantly higher in liver and lung metastases than in locally advanced cancer and metastases to the peritoneum and lymph nodes (Supplemental Fig. S8A,C). This result suggests that peritoneal or lymph node metastasis may not increase the amount of ctDNA leaked from the tumor to blood. In addition, there was no clear correlation between the survival period and VAF (Supplemental Fig. S9C), but patients with detected *KRAS* mutations tended to have shorter survival (Supplemental Fig. S9A,B).

In this study, we demonstrated that multiplex dPCR using melting curve analysis can genotype ctDNA in clinical samples. Furthermore, we achieved a high sensitivity of less than 0.1% for each *KRAS* mutant, except for G13D and G12S. It has been difficult to quantify DNAs and RNAs with both high sensitivity and high multiplexity. Huerta et al. published a systematic review of dPCR analyses of *KRAS* mutations in clinical samples of pancreatic cancer^32^. According to a systematic review, in many cases, multiplexity was limited to the number of probe colors. Even if multiple *KRAS* mutations could be detected simultaneously, the mutation types were not identified. To determine the genotype of *KRAS*, Takai et al. and Taly et al. used probes with different concentrations to improve the multiplicity to five^25,26^. However, the distribution of fluorescence intensity of the multiplexed clusters suggested that further multiplexing was difficult. Calvez-Kelm et al. and Mohan et al. detected *KRAS* mutations in the plasma of patients with advanced pancreatic cancer by NGS^33,34^. Both reports were able to discriminate the mutation type, but the *KRAS* mutation positive rates were 33.3% and 38%, respectively. The rates were lower than those reported using dPCR, probably due to the difference in sensitivity between dPCR and NGS. In this study, 8-plex dPCR with melting curve analysis was used to detect *KRAS* mutations in plasma. Mutations were detectable with high sensitivity in 56.5% of patients with advanced pancreatic cancer, and genotype discrimination was possible at the same time.

Sotorasib has recently been approved as a molecular target drug targeting the G12C mutation, which was found in one clinical specimen measured in this study^19^. Pancreatic ductal adenocarcinoma is caused by somatic alterations in four genes, namely, *KRAS*, *CDKN2A*, *TP53*, and *SMAD4*^35^. There are currently no drugs that target any of these genes except for the G12C mutation in *KRAS*^36^. Although the G12C mutation in the *KRAS* gene is a rare variant found in 1.6% of patients with PDAC, sotorasib yields primarily stable disease when given as a monotherapy in PDAC^37^. In addition to sotorasib, many molecular targeted drugs and vaccines targeting *KRAS* mutations are in development^19^. Since each drug targets a different *KRAS* mutation, it is necessary to identify the mutation type to determine the applicability of the drug. In this study, seven common *KRAS* mutations that are frequently observed in pancreatic and colorectal cancers were genotyped and are expected to be applied to predict the applicability and efficacy of *KRAS*-targeted drugs.

## Methods

### Ethics statement

The experimental protocols were approved by the institutional review board at Hitachi, Ltd. (261-1), the National Cancer Center (2016-053), and Osaka University (19491). Written informed consent was obtained from all patients. The methods were carried out in accordance with the approved guidelines.

### Patients and plasma sample collection

The study involved patients with PDAC diagnosed between 2019 and 2020 at the National Cancer Center Hospital, Tokyo, Japan. Tumors were diagnosed as PDAC based on the histology of resected or biopsied materials. Patients were staged according to the classification of the Union for International Cancer Control (UICC) 8th edition. The clinicopathologic features of the 46 patients in this study are summarized in Supplemental Tables S5 and S6. All peripheral venous blood samples were collected before cancer therapy and immediately processed to isolate plasma by centrifugation in EDTA tubes at 1600×*g* for 10 min at 4 °C. Plasma samples were divided into aliquots and stored at − 80 °C.

*KRAS* mutant genomic DNA reference standards (Horizon Diagnostics, Cambridge, UK) were also used as DNA templates in dPCR.

### Extraction and quantification of cfDNA

Before DNA extraction, plasma samples were centrifuged at 16,000×*g* for 10 min at 4 °C to remove cell debris. Circulating cfDNA was extracted from 10 ml of plasma using a QIAamp MinElute ccfDNA Midi Kit (QIAGEN, Hilden, Germany) according to the manufacturer’s instructions. Circulating cfDNA was eluted into 60 μl of elution buffer and stored at 4 °C. Eluted cfDNA was quantified by a Qubit dsDNA HS Assay Kit (Thermo Fisher Scientific, Massachusetts, USA).

### Quantification of *KRAS* mutations by dPCR combined with melting curve analysis

Absolute quantification of *KRAS* mutations was carried out by dPCR combined with melting curve analysis, with modifications of previously published methods^15,16^. The sequences of primers and probes are listed in Supplemental Table S1. The composition of the reaction solution used for measuring PCR and *T*_m_ in the 8-plex assay in the wells was as follows: 1× QuantStudio 3D Digital PCR Master Mix v2 (Thermo Fisher Scientific), 0.25 μM *KRAS* forward primer, 2.0 μM *KRAS* reverse primer, 0.5 μM *KRAS* wild-type/G13D detection probe, 0.5 μM *KRAS* G12A detection probe, 0.5 μM *KRAS* G12R detection probe, 0.5 μM *KRAS* G12D detection probe, 0.5 μM *KRAS* G12V detection probe, 0.5 μM *KRAS* G12S detection probe, 0.5 μM *KRAS* G12C detection probe, 0.5 μM *KRAS* pseudogene blocker, and 5 μl of cfDNA solution (equivalent to cfDNA from 0.8 ml of plasma) containing a median amount of 6.62 ng (range 2.66–31.32 ng) of cfDNA in a final reaction volume of 15 μl.

After the addition of 14.5 μl of PCR solution to a QuantStudio 3D Digital PCR 20K Chip (Thermo Fisher Scientific), PCR was performed with a thermal cycler. Amplification was carried out as follows: 10 min at 96 °C and 60 cycles of 30 s at 98 °C and 2 min at 60 °C. After PCR, the chip was placed on the temperature control stage of the developed melting temperature measuring device, and three-color fluorescence images of the chip were acquired while increasing the temperature from 50 to 85 °C at 2.0 °C/min. Melting curve analysis of each well was performed from the obtained three-color fluorescence images of the chip, and the *T*_m_ value was calculated.

The cfDNA samples were quantified in triplicate, and the count results of each triplicate sample were merged.

### Quantification of *KRAS* mutations by droplet dPCR

Another absolute quantification of *KRAS* mutations was carried out using the QX200 Droplet Digital PCR System (Bio-Rad, California, USA) and ddPCR *KRAS* Screening Multiplex Kit (Bio-Rad). The composition of the reaction solution used for droplet dPCR was as follows: 1× ddPCR Supermix for Probes (no dUTP) (Bio-Rad), 1× ddPCR *KRAS* Screening Multiplex Assay including primers and probes for seven *KRAS* mutations, and 5 μl of cfDNA (equivalent to cfDNA from 0.8 ml of plasma) in a final reaction volume of 20 μl. Droplet generation, thermal cycling and signal detection were performed according to the manufacturer’s instructions. Data were analyzed using QuantaSoft software v1.7.4.0917 (Bio-Rad).

The cfDNA samples were quantified in triplicate, and the count results of each triplicate sample were merged.

## Supplementary Information

Supplementary Information.

## Data Availability

The datasets used and analyzed during the current study are available from the corresponding author on reasonable request.
