# Supplementary material for: Efficient and accurate KRAS genotyping using digital PCR combined with melting curve analysis for ctDNA from pancreatic cancer patients
Source: Sci Rep. 2023 Feb 21;13:3039. doi: 10.1038/s41598-023-30131-y (PMC9944920; doi:10.1038/s41598-023-30131-y)
Supplement: Supplementary file 1 — Supplementary Information. [file 41598_2023_30131_MOESM1_ESM.pdf]

Supplementary information for

Efficient and accurate *KRAS* genotyping using digital PCR combined with melting curve analysis for ctDNA from pancreatic cancer patients

Junko Tanaka, Tatsuo Nakagawa, Kunio Harada, Chigusa Morizane, Hidenori Tanaka, Satoshi Shiba, Akihiro Ohba, Susumu Hijioka, Erina Takai, Shinichi Yachida, Yoshio Kamura, Takeshi Ishida, Takahide Yokoi, and Chihiro Uematsu

This file includes:

- Supplemental Table S1.
- Supplemental Table S2.
- Supplemental Table S3.
- Supplemental Table S4.
- Supplemental Table S5.
- Supplemental Table S6.
- Supplemental Figure S1.
- Supplemental Figure S2.
- Supplemental Figure S3.
- Supplemental Figure S4.
- Supplemental Figure S5.
- Supplemental Figure S6.
- Supplemental Figure S7.
- Supplemental Figure S8.
- Supplemental Figure S9.

Supplemental Table S1. Sequences of primers, probes and a blocker used for multiplex dPCR assays. All primers were acquired from Integrated DNA Technologies (Iowa, USA), and all probes were acquired from PentaBase (Odense, Denmark). All probes and the blocker contain 4 hydrophobic DNA analogs that prevent degradation during PCR. Mutation positions are underlined.

Primers

Primers	Sequence
Forward Primer	5'-GCTGAAAATGACTGAATATAAACTTGTG-3'
Reverse Primer	5'-GTCAAGGCACTCTTGCCTAC-3'

Probes and blocker

Target	Description	Sequence	Reporter dye	Quencher
Wild type/G13D	Probe	5'-TTGGAGCTGGTGGCGT-3'	HEX	BHQ-1
G12A	Probe	5'-TTGGAGCTG**C**TGGCGT-3'	HEX	BHQ-1
G12R	Probe	5'-TTGGAGCT**C**GTGGCGT-3'	FAM	BHQ-1
G12D	Probe	5'-TGGAGCTG**A**TGGCGT-3'	FAM	BHQ-1
G12V	Probe	5'-GAGCTG**T**TGGCGT-3'	FAM	BHQ-1
G12S	Probe	5'-TGGAGCT**A**GTGGCGT-3'	Cy5	BHQ-2
G12C	Probe	5'-GAGCT**T**GTGGCGT-3'	Cy5	BHQ-2
Pseudogene	Blocker	5'-TTGGAGCTGGT**A**GCGT-3'	-	-

Supplemental Table S2. dPCR counts for each mutant from wild-type control cfDNAs. Total counts (double-positive counts). Double positives of G12A and G13D were not able to be counted, because their double positives were counted only when the numbers of single positives were above the threshold.

Sample number	Wild type	G12R	G12D	G12V	G12A	G13D	G12S	G12C
1	5033	0 (0)	0 (0)	1 (0)	0 (-)	2 (-)	7 (7)	0 (0)
2	4919	0 (0)	0 (0)	1 (0)	0 (-)	1 (-)	0 (0)	0 (0)
3	4926	0 (0)	0 (0)	0 (0)	0 (-)	4 (-)	0 (0)	0 (0)
4	4829	0 (0)	0 (0)	0 (0)	0 (-)	4 (-)	3 (3)	0 (0)
5	5046	0 (0)	1 (0)	0 (0)	1 (-)	2 (-)	1 (1)	0 (0)
6	4826	0 (0)	2 (2)	0 (0)	2 (-)	1 (-)	2 (2)	1 (1)
7	4671	1 (1)	1 (0)	0 (0)	0 (-)	0 (-)	1 (1)	0 (0)
8	3011	0 (0)	1 (1)	0 (0)	0 (-)	1 (-)	0 (0)	0 (0)

Supplemental Table S3. VAF of KRAS mutations in plasma cfDNA detected by conventional dPCR and dPCR with melting curve analysis.

Patient ID	Conventional dPCR	dPCR with melting curve analysis						
		G12R	G12D	G12V	G12A	G13D	G12S	G12C
#01	0.18%	0.00%	0.08%	0.15%	0.08%	0.00%	0.15%	0.00%
#02	0.39%	0.00%	0.42%	0.00%	0.00%	0.00%	0.00%	0.00%
#03	3.76%	0.00%	4.06%	0.00%	0.00%	0.02%	0.04%	0.02%
#04	5.66%	0.00%	0.08%	4.96%	0.00%	0.03%	0.06%	0.00%
#05	3.22%	0.00%	3.45%	0.00%	0.00%	0.14%	0.04%	0.00%
#06	0.08%	0.01%	0.01%	0.02%	0.01%	0.01%	0.03%	0.01%
#07	0.21%	0.00%	0.07%	0.00%	0.00%	0.07%	0.00%	0.00%
#08	1.02%	0.00%	0.07%	1.10%	0.00%	0.00%	0.07%	0.00%
#09	0.04%	0.00%	0.04%	0.04%	0.00%	0.00%	0.00%	0.00%
#10	0.35%	0.02%	0.03%	0.14%	0.02%	0.05%	0.03%	0.02%
#11	23.18%	0.00%	23.56%	0.03%	0.00%	0.03%	0.00%	0.03%
#12	0.04%	0.00%	0.06%	0.00%	0.00%	0.06%	0.00%	0.06%
#13	0.04%	0.00%	0.00%	0.00%	0.00%	0.11%	0.15%	0.07%
#14	13.33%	0.00%	12.69%	0.00%	0.00%	0.10%	0.19%	0.03%
#15	0.00%	0.00%	0.14%	0.00%	0.00%	0.03%	0.14%	0.00%
#16	0.07%	0.00%	0.03%	0.00%	0.00%	0.00%	0.05%	0.08%
#17	0.14%	0.00%	0.07%	0.00%	0.00%	0.00%	0.10%	0.00%
#18	1.54%	0.00%	1.41%	0.00%	0.00%	0.00%	0.08%	0.00%
#19	0.05%	0.04%	0.06%	0.00%	0.00%	0.04%	0.12%	0.00%
#20	2.28%	0.00%	0.00%	2.39%	0.00%	0.11%	0.11%	0.16%
#21	0.06%	0.00%	0.00%	0.00%	0.00%	0.09%	0.00%	0.00%
#22	1.68%	0.00%	1.51%	0.00%	0.00%	0.03%	0.20%	0.06%
#23	0.05%	0.02%	0.02%	0.02%	0.00%	0.02%	0.13%	0.04%
#24	0.00%	0.00%	0.00%	0.00%	0.03%	0.00%	0.20%	0.00%
#25	0.00%	0.00%	0.08%	0.00%	0.00%	0.00%	0.18%	0.06%
#26	0.00%	0.00%	0.03%	0.00%	0.03%	0.08%	0.08%	0.00%
#27	0.00%	0.00%	0.07%	0.00%	0.07%	0.00%	0.13%	0.00%
#28	45.38%	0.00%	0.04%	44.86%	0.03%	0.10%	0.06%	0.05%
#29	0.26%	0.00%	0.03%	0.00%	0.00%	0.17%	0.17%	0.03%
#30	0.15%	0.00%	0.05%	0.00%	0.00%	0.05%	0.05%	0.05%
#31	0.58%	0.00%	0.64%	0.02%	0.00%	0.02%	0.08%	0.02%
#32	0.05%	0.00%	0.01%	0.00%	0.01%	0.00%	0.04%	0.01%
#33	0.57%	0.00%	0.11%	0.25%	0.00%	0.00%	0.05%	0.00%
#34	0.06%	0.00%	0.05%	0.00%	0.01%	0.04%	0.17%	0.02%
#35	14.23%	0.00%	14.23%	0.01%	0.00%	0.05%	0.12%	0.01%
#36	0.03%	0.02%	0.02%	0.00%	0.02%	0.09%	0.11%	0.02%
#37	5.57%	0.00%	0.03%	6.10%	0.06%	0.07%	0.06%	0.01%
#38	0.16%	0.00%	0.21%	0.00%	0.00%	0.00%	0.04%	0.02%
#39	0.16%	0.00%	0.24%	0.00%	0.03%	0.00%	0.03%	0.00%
#40	0.69%	0.00%	0.02%	0.00%	0.01%	0.05%	0.07%	0.79%
#41	0.35%	0.00%	0.00%	0.26%	0.13%	0.00%	0.00%	0.00%
#42	0.28%	0.01%	0.00%	0.11%	0.02%	0.01%	0.23%	0.02%
#43	0.03%	0.00%	0.00%	0.00%	0.00%	0.02%	0.08%	0.04%
#45	0.59%	0.00%	0.58%	0.00%	0.00%	0.00%	0.08%	0.03%
#46	0.07%	0.00%	0.00%	0.00%	0.00%	0.00%	0.11%	0.00%
#47	0.83%	0.00%	0.02%	0.62%	0.00%	0.02%	0.11%	0.00%

Locally advanced
(including early-stage cancers (#2 and #41) and local recurrence after surgery (#15 and #26))

Peritoneal or lymph node metastasis

Liver or lung metastasis
(including metastasis after surgery (#08, #20 and #28))

Highest VAF

Supplemental Table S4. KRAS mutation copies in plasma cfDNA detected by conventional dPCR and dPCR with melting curve analysis. Total counts (double positive counts).

Patient ID	Conventional dPCR	dPCR with melting analysis						
		G12R	G12D	G12V	G12A	G13D	G12S	G12C
#01	4	0 (0)	1 (1)	2 (0)	1 (0)	0 (0)	2 (2)	0 (0)
#02	4	0 (0)	3 (1)	0 (0)	0 (0)	0 (0)	0 (0)	0 (0)
#03	266	0 (0)	187 (17)	0 (0)	0 (0)	1 (0)	2 (2)	1 (1)
#04	276	0 (0)	3 (1)	177 (11)	0 (0)	1 (0)	2 (1)	0 (0)
#05	142	0 (0)	97 (4)	0 (0)	0 (0)	4 (0)	1 (1)	0 (0)
#06	11	1 (1)	1 (1)	2 (0)	1 (0)	1 (0)	3 (3)	1 (0)
#07	9	0 (0)	2 (0)	0 (0)	0 (0)	2 (0)	0 (0)	0 (0)
#08	60	0 (0)	3 (3)	47 (1)	0 (0)	0 (0)	3 (3)	0 (0)
#09	2	0 (0)	2 (2)	2 (0)	0 (0)	0 (0)	0 (0)	0 (0)
#10	33	1 (1)	2 (2)	9 (1)	1 (0)	3 (0)	2 (2)	1 (0)
#11	996	0 (0)	689 (41)	1 (0)	0 (0)	1 (0)	0 (0)	1 (0)
#12	2	0 (0)	2 (2)	0 (0)	0 (0)	2 (0)	0 (0)	2 (0)
#13	2	0 (0)	0 (0)	0 (0)	0 (0)	3 (0)	4 (4)	2 (2)
#14	572	0 (0)	394 (33)	0 (0)	0 (0)	3 (0)	6 (6)	1 (1)
#15	0	0 (0)	4 (3)	0 (0)	0 (0)	1 (0)	4 (4)	0 (0)
#16	4	0 (0)	1 (1)	0 (0)	0 (0)	0 (0)	2 (2)	3 (2)
#17	6	0 (0)	2 (1)	0 (0)	0 (0)	0 (0)	3 (3)	0 (0)
#18	60	0 (0)	37 (4)	0 (0)	0 (0)	0 (0)	2 (2)	0 (0)
#19	3	2 (0)	3 (3)	0 (0)	0 (0)	2 (0)	6 (5)	0 (0)
#20	56	0 (0)	0 (0)	45 (2)	0 (0)	2 (0)	2 (2)	3 (1)
#21	2	0 (0)	0 (0)	0 (0)	0 (0)	2 (0)	0 (0)	0 (0)
#22	86	0 (0)	52 (5)	0 (0)	0 (0)	1 (0)	7 (6)	2 (1)
#23	4	1 (1)	1 (1)	1 (0)	0 (0)	1 (0)	6 (6)	2 (1)
#24	0	0 (0)	0 (0)	0 (0)	1 (0)	0 (0)	6 (5)	0 (0)
#25	0	0 (0)	5 (4)	0 (0)	0 (0)	0 (0)	11 (11)	4 (3)
#26	0	0 (0)	1 (1)	0 (0)	1 (0)	3 (0)	3 (3)	0 (0)
#27	0	0 (0)	1 (0)	0 (0)	1 (0)	0 (0)	2 (2)	0 (0)
#28	17860	0 (0)	10 (2)	11491 (2882)	7 (0)	26 (0)	15 (15)	13 (5)
#29	10	0 (0)	1 (0)	0 (0)	0 (0)	5 (0)	5 (5)	1 (1)
#30	4	0 (0)	1 (1)	0 (0)	0 (0)	1 (0)	1 (0)	1 (1)
#31	41	0 (0)	32 (1)	1 (0)	0 (0)	1 (0)	4 (3)	1 (1)
#32	23	0 (0)	3 (3)	0 (0)	2 (0)	0 (0)	16 (16)	3 (3)
#33	28	0 (0)	4 (3)	9 (0)	0 (0)	0 (0)	2 (1)	0 (0)
#34	8	0 (0)	5 (5)	0 (0)	1 (0)	4 (0)	16 (16)	2 (2)
#35	1526	0 (0)	1066 (167)	1 (0)	0 (0)	4 (0)	9 (9)	1 (1)
#36	2	1 (1)	1 (1)	0 (0)	1 (0)	4 (0)	5 (5)	1 (0)
#37	542	0 (0)	2 (1)	410 (88)	4 (0)	5 (0)	4 (4)	1 (0)
#38	11	0 (0)	11 (2)	0 (0)	0 (0)	0 (0)	2 (2)	1 (0)
#39	8	0 (0)	8 (1)	0 (0)	1 (0)	0 (0)	1 (1)	0 (0)
#40	140	0 (0)	3 (2)	0 (0)	2 (0)	7 (0)	10 (9)	111 (30)
#41	8	0 (0)	0 (0)	4 (0)	2 (0)	0 (0)	0 (0)	0 (0)
#42	37	1 (0)	0 (0)	11 (1)	2 (0)	1 (0)	23 (22)	2 (1)
#43	2	0 (0)	0 (0)	0 (0)	0 (0)	1 (0)	4 (4)	2 (2)
#45	31	0 (0)	21 (4)	0 (0)	0 (0)	0 (0)	3 (3)	1 (0)
#46	4	0 (0)	0 (0)	0 (0)	0 (0)	0 (0)	4 (4)	0 (0)
#47	50	0 (0)	1 (1)	27 (3)	0 (0)	1 (0)	5 (5)	0 (0)

[white box] Locally advanced (including early-stage cancers (#2 and #41) and local recurrence after surgery (#15 and #26))

[green box] Peritoneal or lymph node metastasis

[yellow box] Liver or lung metastasis (including metastasis after surgery (#08, #20 and #28))

[blue box] Largest number of mutation copies

Supplemental Table S5. Clinicopathological data.

	n	%
Gender		
Male	23	50.0
Female	23	50.0
Age (Years)		
Mean	64.6	
Median	65.5	
Range	40 - 85	
Tumor location		
Head	25	54.3
Body/tail	21	45.7
Tumor size (mm)		
Mean	36.3	
Median	35.0	
Range	14 - 75	
T factor (UICC)		
T1	1	2.2
T2	12	26.1
T3	7	15.2
T4	21	45.7
N factor (UICC)		
N0	19	41.3
N1	16	34.8
N2	6	13.0
M factor (UICC)		
M0	15	32.6
M1	26	56.5
UICC stage		
I	1	2.2
II	1	2.2
III	13	28.3
IV	26	56.5
Recurrence		
Local	2	4.3
Distant organ metastasis	3	6.5

Supplemental Table S6. Clinicopathological data of each patient.

Patient ID	Stage	Recurrent	Metastasis				
			Lymph node	Peritoneal	Liver	Lung	Bone
#01	III	-					
#02	II	-					
#03	IV	-			+	+	+
#04	IV	-			+	+	
#05	IV	-		+		+	
#06	III	-					
#07	IV	-		+			
#08		+	+		+	+	
#09	III	-					
#10	IV	-			+		
#11	IV	-	+		+		
#12	IV	-	+	+	+		
#13	IV	-	+			+	
#14	IV	-			+		
#15		+					
#16	IV	-	+	+			
#17	III	-					
#18	III	-					
#19	III	-					
#20		+			+		
#21	IV	-		+			
#22	IV	-	+				
#23	IV	-		+			
#24	III	-					
#25	IV	-		+			
#26		+					
#27	III	-					
#28		+	+	+	+		
#29	III	-					
#30	IV	-		+			
#31	III	-					
#32	III	-					
#33	IV	-	+		+		
#34	IV	-	+	+			
#35	IV	-	+		+		
#36	III	-					
#37	IV	-	+	+			
#38	IV	-	+				
#39	III	-					
#40	IV	-	+	+	+		
#41	I	-					
#42	IV	-	+		+		
#43	IV	-	+	+			
#45	IV	-	+				
#46	IV	-	+		+		
#47	IV	-	+		+		

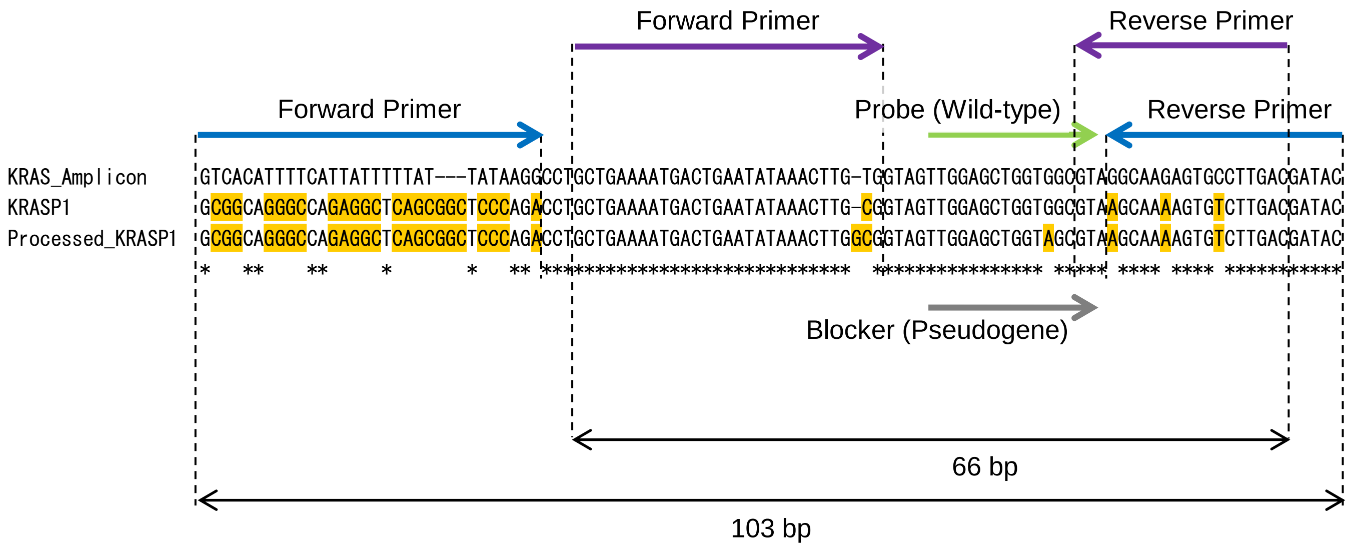

Supplemental Figure S1. Alignment of *KRAS* and two types of *KRAS* pseudogenes. Mismatch sites between *KRAS* and the *KRAS* pseudogene are highlighted in yellow. Blue arrows indicate conventional primers used in the previous report (Nakagawa *et al*., *Anal Chem*, 2020), and violet arrows indicate new primers used in this study. Green arrow indicates a probe for wild-type *KRAS.* Gray arrow indicates a blocker for the *KRAS* pseudogene processed *KRASP1*.

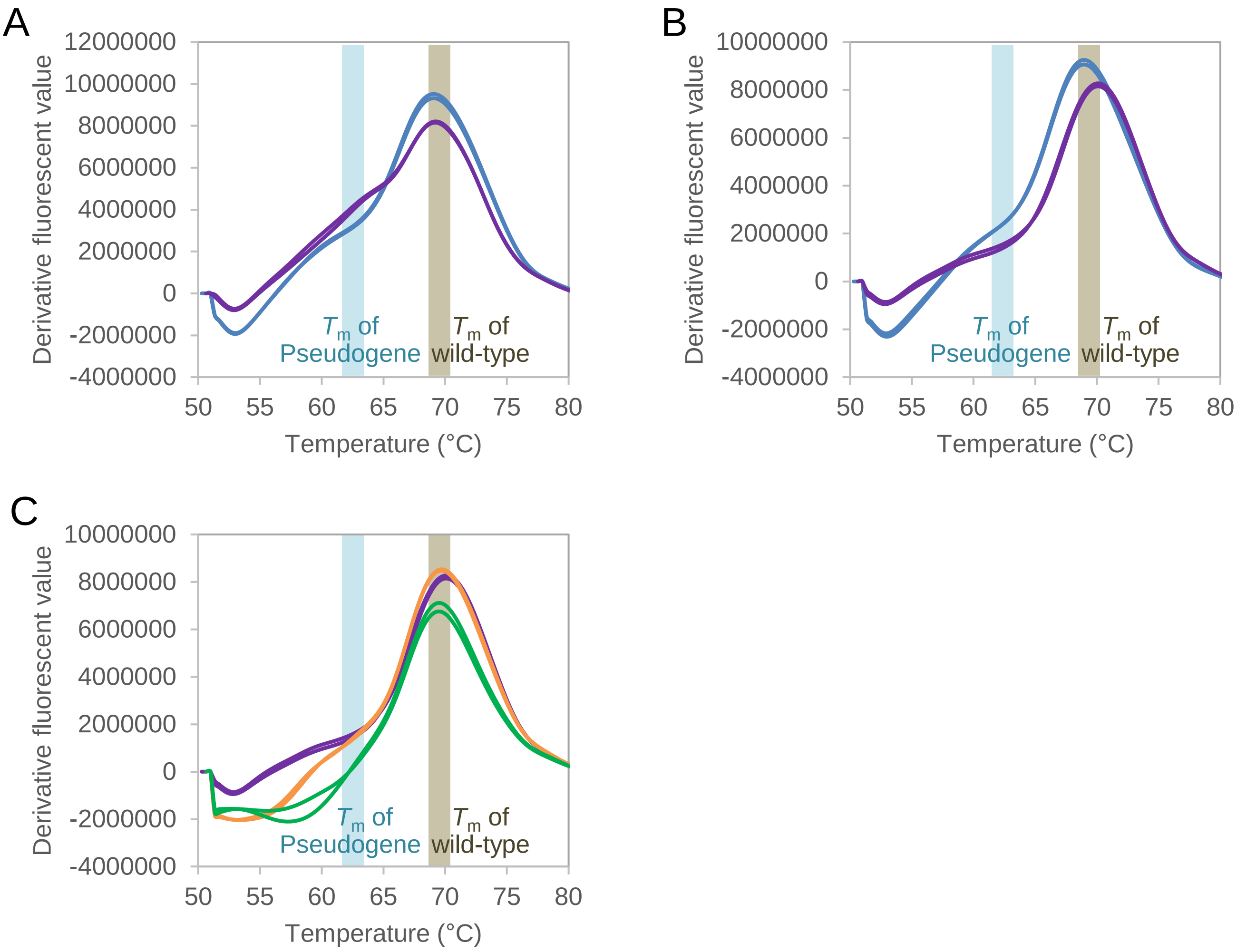

Supplemental Figure S2. Melting curve analysis using conventional and new primers. (A), (B) Melting curve analysis was performed after PCR with conventional and new primers using genomic DNA as a template. Blue, conventional primers; violet, new primers. Annealing temperatures were 55 °C in (A), and 60 °C in (B). (C) Melting curve analysis was performed after PCR with new primers and a blocker for *KRAS* pseudogenes. Violet, 0.0 µM blocker; orange, 0.5 µM blocker; green, 2.0 µM blocker.

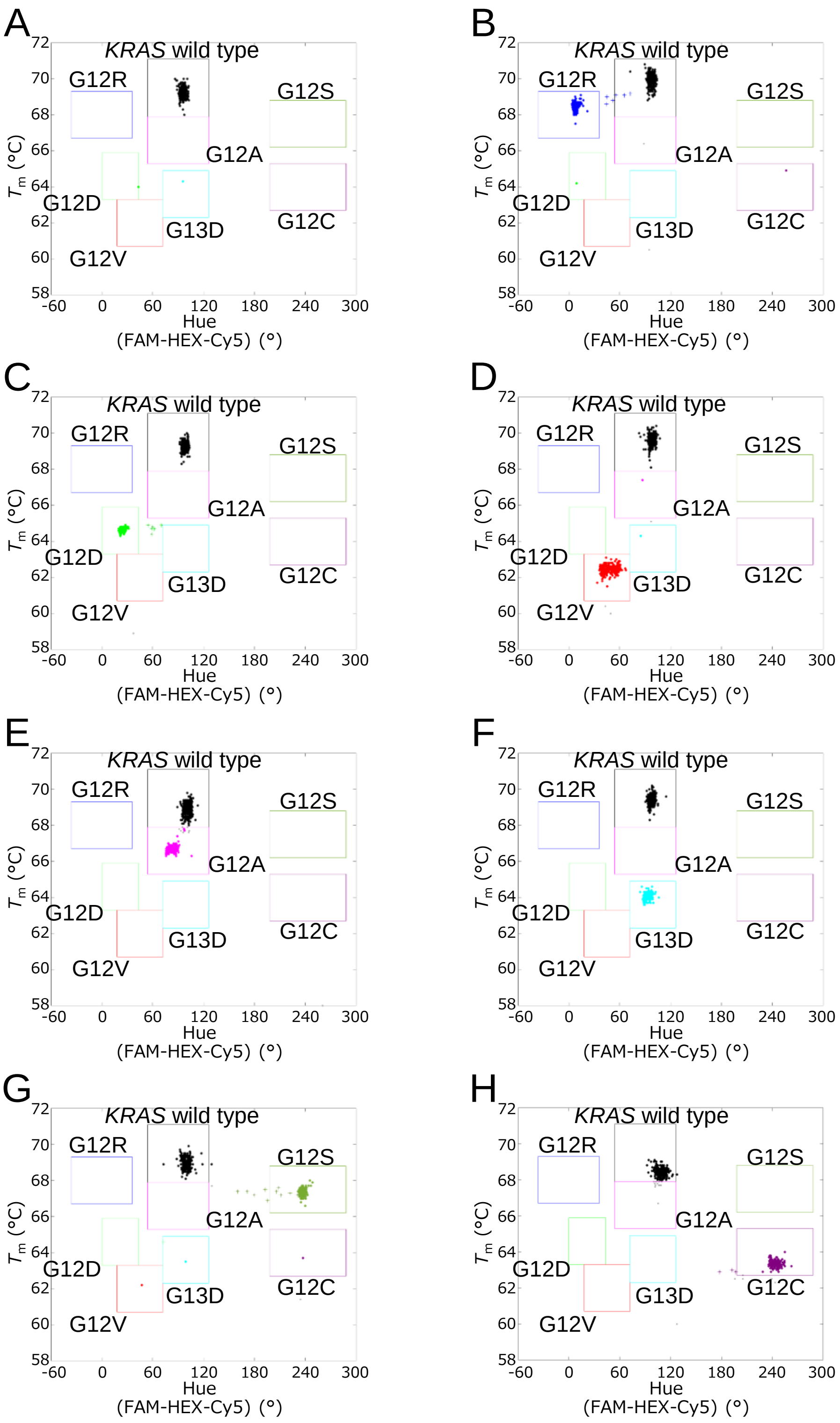

Supplemental Figure S3. Genotyping results of the proposed multiplex assay for each genome standard sample. (A) Wild-type, (B) G12R, (C) G12D, (D) G12V, (E) G12A, (F) G13D, (G) G12S, and (H) G12C. Note that each standard sample of a mutant includes the wild-type and one mutant. Dots indicate a single-positive well of wild-type or mutant; plus signs indicate a double-positive well of wild-type and one of the mutants.

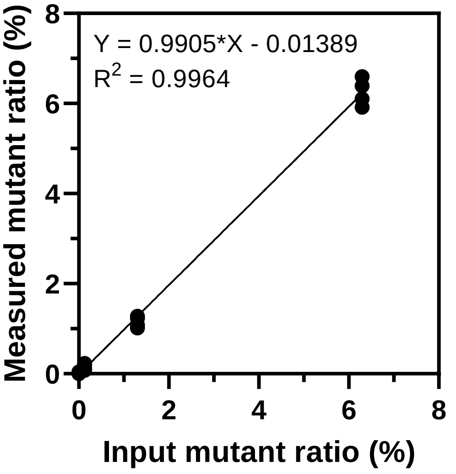

Supplemental Figure S4. Quantification results for the measured mutant ratio as a function of the input mutant ratio obtained with the *KRAS* G12D cfDNA standard samples. N=4 for each input mutant ratio.

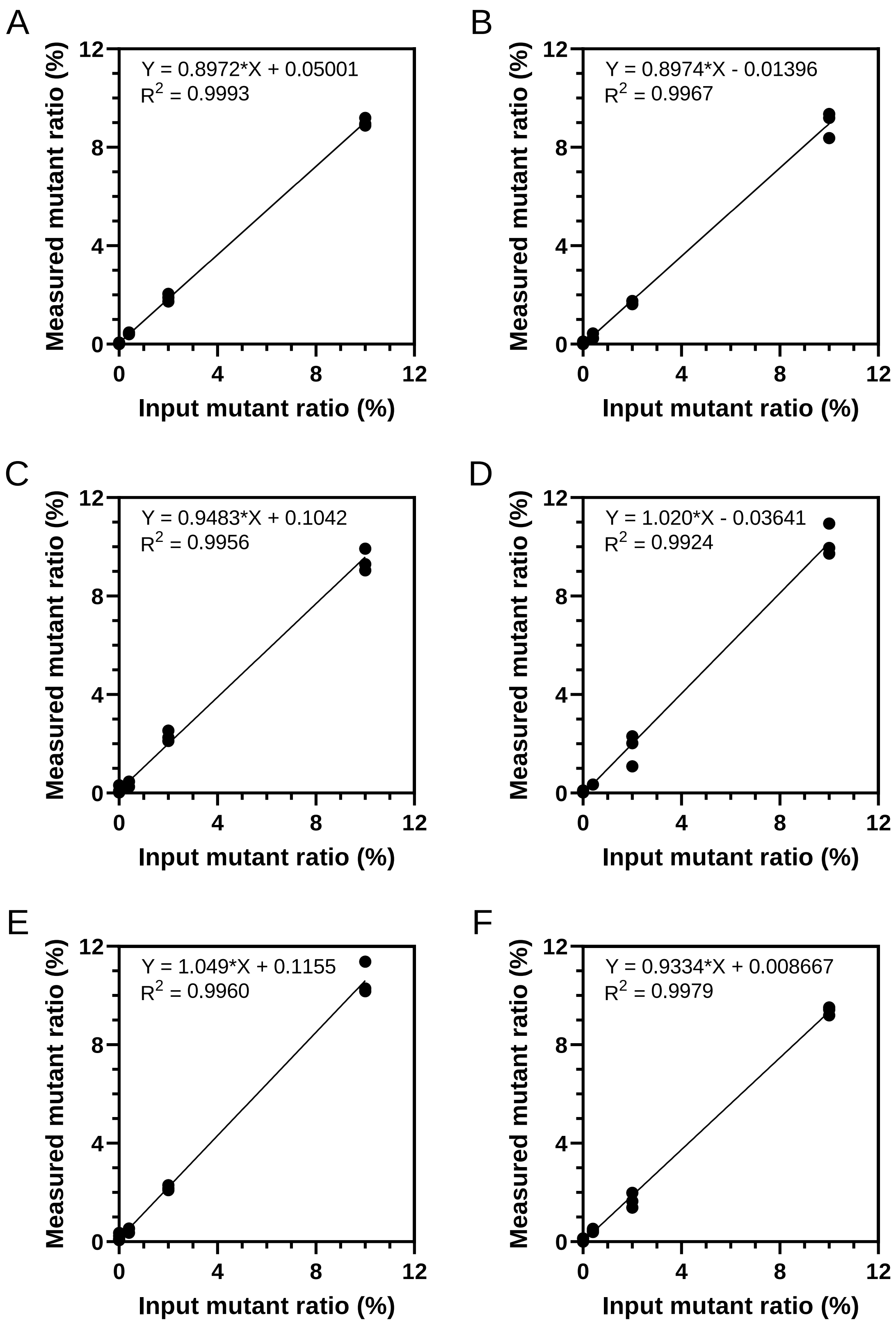

Supplemental Figure S5. Quantification results for the measured mutant ratio as a function of the input mutant ratio obtained with (A) G12R, (B) G12V, (C) G12A, (D) G13D, (E) G12S, and (F) G12C fragmented gDNA spiked into wild-type fragmented gDNA. N=3 for each input mutant ratio.

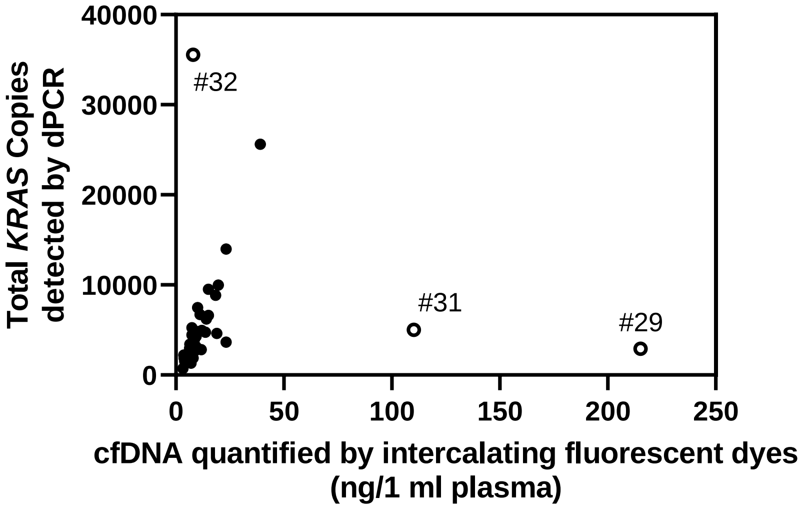

Supplemental Figure S6. The relationship between the cfDNA concentration measured by intercalating fluorescent dyes and the total *KRAS* copies detected by dPCR with melting curve analysis. The amount of cfDNA correlated with the copy number of the *KRAS* gene in all but three samples. Samples #29 and #31, which showed cfDNA concentrations exceeding 100 ng/ml, were highly hemolytic and were likely contaminated with reticulocyte RNA or leukocyte DNA during blood collection and plasma separation. In sample #32, the copy number of the *KRAS* gene was high compared to the amount of cfDNA, suggesting that *KRAS* gene amplification occurred during carcinogenesis. The mean and median amounts of cfDNA in 44 samples (excluding #29 and #31) were 10.59±6.55 ng/ml and 8.28 ng/ml, respectively. The amounts of cfDNA are known to vary depending on cancer type, cancer stage, and the presence of metastasis.[2,3,5] In particular, samples from patients with colorectal cancer have been reported to have higher cfDNA concentrations than samples from patients with other cancers, with a mean value of 66 ng/ml in stage IV and 21 ng/ml in stages I-III.[3] On the other hand, the amount of cfDNA in PDAC differs among reports. Takai *et al*. analyzed the cfDNA of PDAC patients and reported that the median amounts of cfDNA in stage IV and stage I-III patients were 10.83 ng/ml and 8.80 ng/ml, respectively.[26] In contrast, two reports showed higher amounts of cfDNA in PDAC patients, i.e., 100±5 ng/ml and 99.2±137.2 ng/ml.[30,33] The cfDNA levels in this study were in approximate agreement with those reported by Takai *et al*. and supported the result that cfDNA levels in PDAC patients were lower than those in other cancer types.

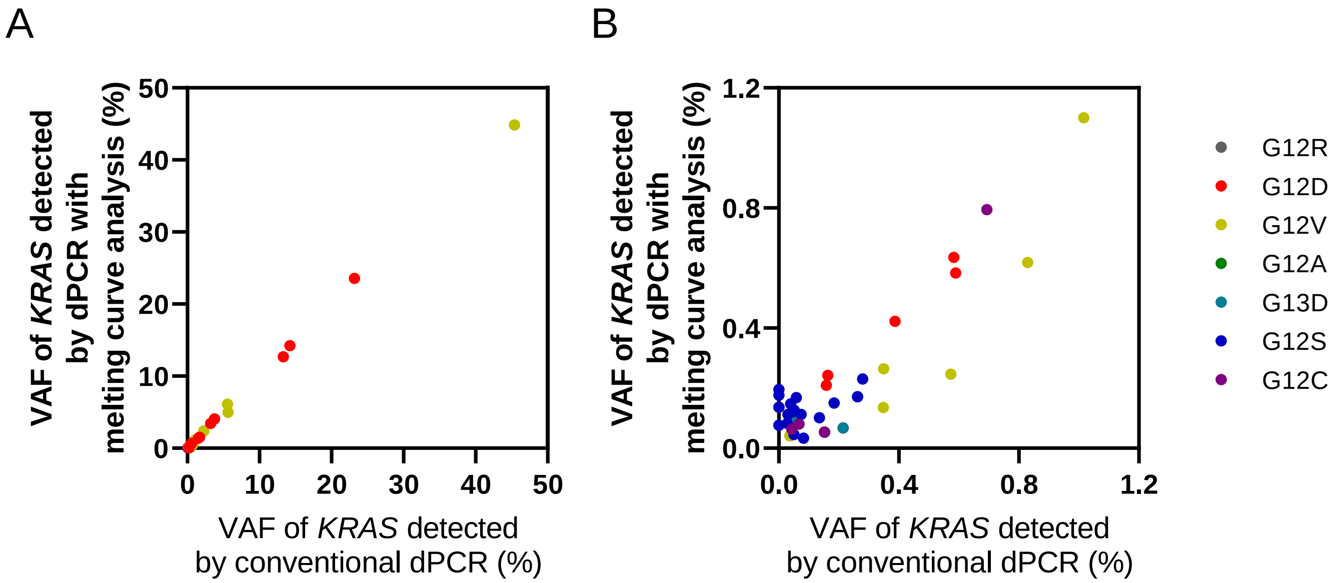

Supplemental Figure S7. Comparison between the percentage of variant allele frequency (VAF) of *KRAS* detected by dPCR with melting curve analysis and conventional dPCR. Only the genotype with the highest VAF in each clinical sample was plotted. (B) shows the enlarged low VAF data of (A).

Supplemental Figure S7B, which is an enlargement of the region with low VAFs, shows that G12S is frequently detected with a VAF <0.4%, which is considered to be a false positive due to PCR error. In Supplemental Table S5, the genotype with the highest VAF in each clinical sample is highlighted in blue when its VAF is higher than the LOD of the respective genotype shown in Table 1. Conventional dPCR results were highlighted in blue when the VAF was higher than 0.2%, the LOD of the kit, according to the instructions of the kit. In samples #24 and #25, G12S mutants were detected by dPCR using melting curve analysis, but they were almost all double-positive wells for the wild-type and G12S (Supplemental Table S6). Therefore, they were considered false positives due to PCR error. Although both G12V and G12S were detected in sample #42, the G12S wells were probably false positives, and only G12V was truly positive because almost all of the G12S samples detected were double-positive wells with the wild-type and G12S. The most commonly detected mutant genotypes for the measured samples were G12D or G12V except for sample #29 and #40, where G13D and G12C were detected. According to the COSMIC database, mutations in codons 12 and 13 of the *KRAS* gene in pancreatic cancer samples are 50% G12D and 30% G12V, which is nearly the same as our results, in which 54%, 40%, 4% and 4% were detected for G12D, G12V, G13D, and G12C, respectively.

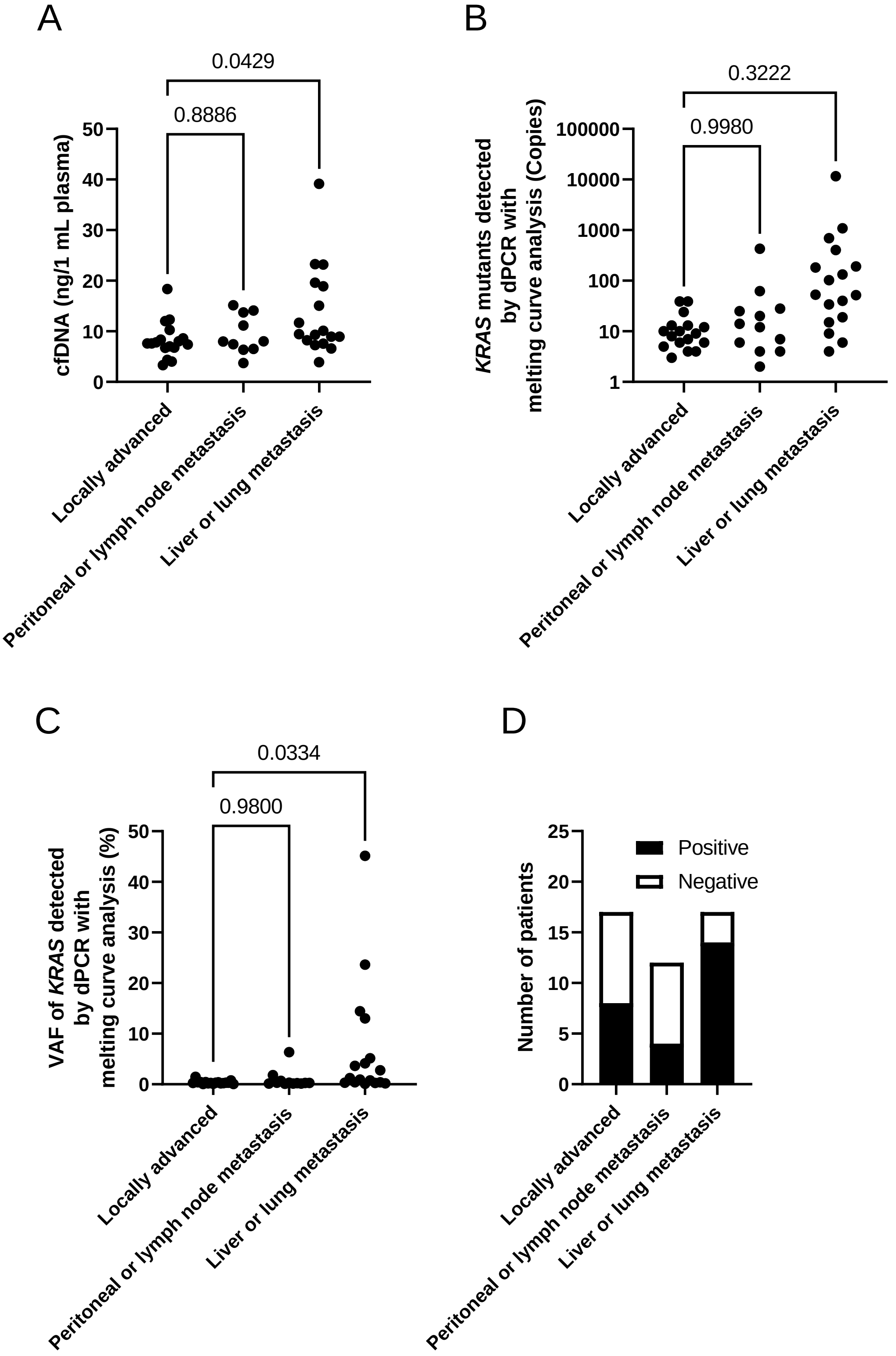

Supplemental Figure S8. The relationship between the detectability of mutant KRAS in plasma cell-free DNA (cfDNA) and distant organ metastasis at diagnosis. The relationships between the amount of cfDNA (A), the copy number of mutant *KRAS* (B), the VAF of mutant *KRAS* (C), and the number of patients with or without detectable levels of *KRAS* mutations (D) and distant organ metastasis at diagnosis.

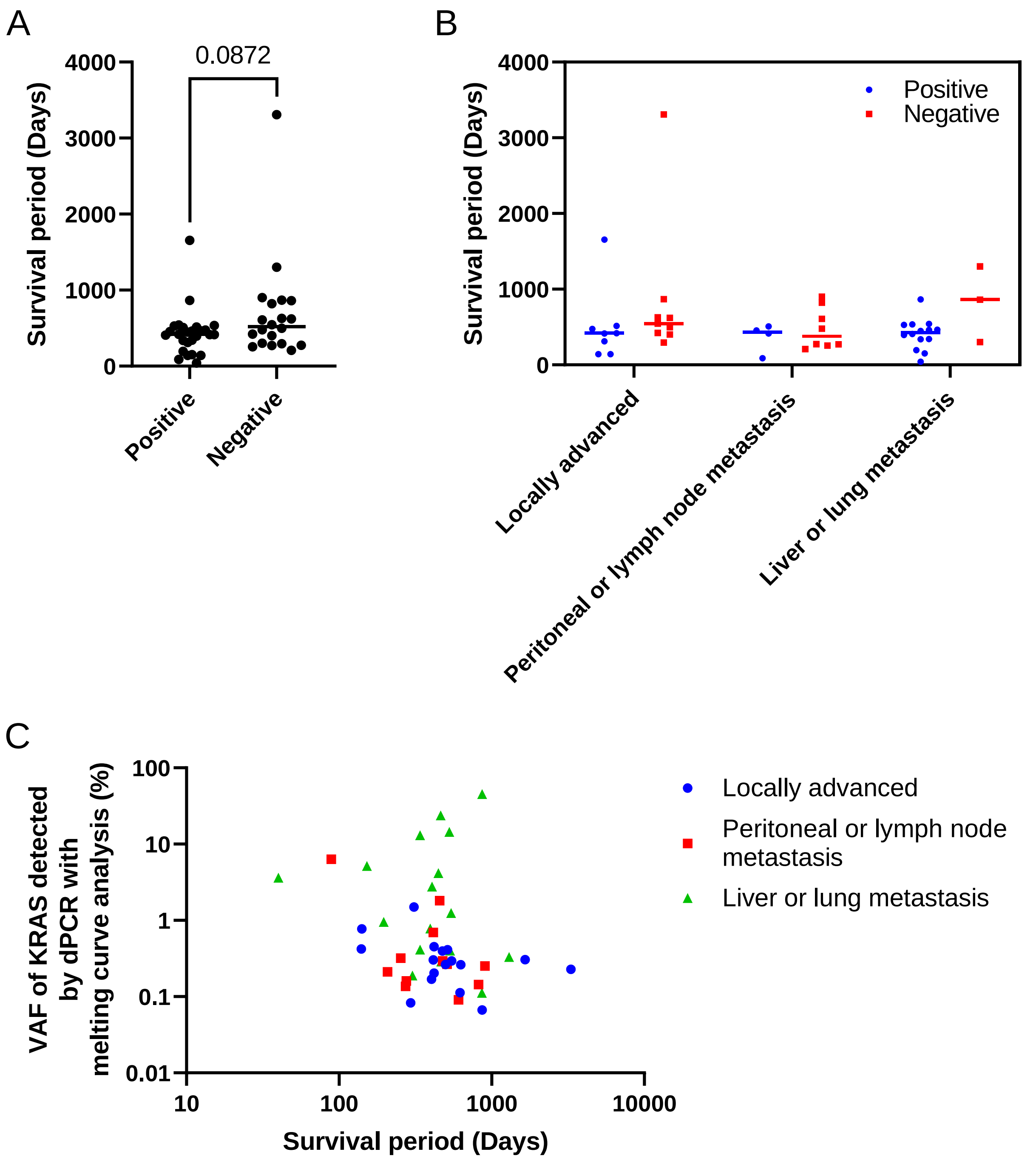

Supplemental Figure S9. The relationship between the detectability of mutant *KRAS* in plasma cfDNA and survival period. (A), (B) The relationship between detectability of mutant *KRAS* in plasma cfDNA and survival period in whole samples (A) and in each metastatic status (B). (C) The relationship between survival period and VAF of *KRAS* mutations in plasma cfDNA.
